# Neuroimaging evaluations of olfactory, gustatory, and neurological deficits in patients with long-term sequelae of COVID-19

**DOI:** 10.1007/s11682-024-00936-0

**Published:** 2024-09-28

**Authors:** Carla Masala, Michele Porcu, Gianni Orofino, Giovanni Defazio, Ilenia Pinna, Paolo Solla, Tommaso Ercoli, Jasjit S. Suri, Giacomo Spinato, Luca Saba

**Affiliations:** 1 Department of Biomedical Sciences, University of Cagliari, SP8 Cittadella Universitaria Monserrato, Monserrato, Cagliari, 09042 Italy; 2 Department of Radiology, AOU Cagliari, University of Cagliari, SS 554 km 4.500, Cagliari, 09042 Italy; 3 Department of Neurology, AOU Cagliari, University of Cagliari, SS 554 km 4.500, Cagliari, 09042 Italy; 4 Department of Translational Biomedicine and Neuroscience, University of Bari, Bari, 70121 Italy; 5 Neurological Unit, AOU Sassari, University of Sassari, Viale S. Pietro 10, Sassari, 07100 Italy; 6 Department of ECE, Idaho State University, Pocatello, ID 83209 USA; 7 Department of CE, Graphics Era Deemed to be University, Dehradun, 248002 India; 8 University Center for Research & Development, Chandigarh University, Mohali, India; 9 Symbiosis Institute of TechnologySymbiosis International (Deemed University), Nagpur Campus, Pune, India; 10 Stroke Diagnostic and Monitoring Division, AtheroPoint™, Roseville, CA 95661 USA; 11 Department of Neurosciences, Otolaryngology Unit, University of Padova, Padova, 35100 Italy

**Keywords:** Neuroimaging, olfactory dysfunction, COVID-19, Anosmia, Ageusia, Hyposmia

## Abstract

The World Health Organization indicated that around 36 million of patients in the European Region showed long COVID associated with olfactory and gustatory deficits. The precise mechanism underlying long COVID clinical manifestations is still debated. The aim of this study was to evaluate potential correlations between odor threshold, odor discrimination, odor identification, and the activation of specific brain areas in patients after COVID-19. Sixty subjects, 27 patients (15 women and 12 men) with long COVID and a mean age of 40.6 ± 13.4 years, were compared to 33 age-matched healthy controls (20 women and 13 men) with a mean age of 40.5 ± 9.8 years. Our data showed that patients with long COVID symptoms exhibited a significant decrease in odor threshold, odor discrimination, odor identification, and their sum TDI score compared to age-matched healthy controls. In addition, our results indicated significant correlations between odor discrimination and the increased activation in the right hemisphere, in the frontal pole, and in the superior frontal gyrus. This study indicated that the resting-state fMRI in combination with the objective evaluation of olfactory and gustatory function may be useful for the evaluation of patients with long COVID associated with anosmia and hyposmia.

**Supplementary Information:**

The online version contains supplementary material available at 10.1007/s11682-024-00936-0.

## Introduction

The advent of the SARS-CoV-2 virus, responsible for the Coronavirus Disease 2019 (COVID-19), has amplified public awareness regarding the implications of olfactory dysfunction in daily living. The olfactory epithelium is usually exposed to damage from toxic agents and a host of viruses, such as COVID-19 (Doty et al. 2022). The most common symptoms associated with COVID-19 were olfactory and gustatory dysfunctions (Whitcroft & Hummel, 2020; Xu et al., 2022; Spinato et al., 2020). Patients with neurological deficits such as anosmia and/or ageusia were 17 times more likely to test positive for COVID-19 compared to those without any symptoms (Doty et al. 2022). Gustatory deficits among COVID-19 patients could potentially be indicative of olfactory dysfunction, given the pivotal contribution of retro-nasal olfaction to our perception of flavor. The time for the recovery of function after symptoms of olfactory impairment is around 10 days, however there are some subjects with persistent symptoms for more than 3 months following the acute phase, called long COVID (Xydakis et al., 2021). Many patients showed long COVID symptoms associated with the nervous system, such as olfactory and gustatory deficits, brain fog, cognitive dysfunction, and fatigue (Stefanou et al., 2022). According to the World Health Organization, around 36 million patients in the European region may have experienced long COVID in the first 3 years of the pandemic, and around 1 in 30 may still be finding it hard to return to normal life.

Different hypotheses have been proposed to explain the mechanism of these long COVID symptoms associated with the nervous system, however the neuroinvasion mechanism related to SARS-CoV-2 is not yet well known (Tai et al., 2023). A recently published study showed that around 35% of patients who had a COVID-19 acute infection showed the persistence of qualitative disturbances of smell and/or taste (Ercoli et al., 2021). Various mechanisms have been proposed to elucidate how the SARS-CoV-2 virus might infiltrate the central nervous system. Previous studies hypothesized and demonstrated that the SARS-CoV-2 may enter the brain and cross the blood brain barrier using olfactory retrograde axonal transport and the enteric nervous system (Ousseiran et al. 2023; Zhang et al., 2021).

COVID-19-induced olfactory dysfunction is usually connected to structural and functional changes of the brain areas (Tai et al., 2023). The most common brain degenerations involved in olfactory dysfunctions are the decreased volume of the olfactory bulb (Altunisik et al., 2021; Frosolini et al., 2022), the decreased gray matter volume in olfactory brain areas (such as orbitofrontal cortex, piriform cortex, amygdala, insula, and anterior cingulate) (Nigri et al., 2013), the reduced connections among different brain areas (Seubert et al., 2013), and damage to limbic system (Thomasson et al., 2023). The most common neurological symptoms of COVID-19 are headache, dizziness, stroke, encephalitis, acute myelitis, and encephalopathy in the central nervous system; anosmia/ageusia in the peripheral nervous system; myalgia and myasthenia gravis in skeletal muscle manifestations (Ousseiran et al. 2023).

A previous study evaluated olfactory and gustatory function eight months after acute COVID-19 using self-reported and psychophysical tests to quantify the prevalence of hyposmia and hypogeusia in post-COVID-19 patients without any resting-state fMRI (rs-fMRI) (Hintschich et al., 2022). In our study, were combined objective analyses for the evaluation of olfactory and gustatory function using Sniffin’ Sticks, Taste Strips tests, and resting-state fMRI (rs-fMRI) analyses performed with the fractional Amplitude of Low Frequency Fluctuations (fALFF) method, a research technique extensively used for analyzing regional neural activity (Lv et al., 2018; Zou et al., 2008).

The aim of this study was to determine any potential correlations between odor threshold, odor discrimination, odor identification, and the activation of specific brain areas in patients after COVID-19. First, our attention focused on the evaluation of significant differences between patients with long COVID symptoms and age-matched healthy controls for the following parameters: odor threshold, odor discrimination, odor identification, sweet, salty, sour, and bitter taste perception, depression level, and cognitive abilities. Then, our goal was to analyse the correlation between the olfactory test performance in patients with long COVID symptoms and the volume of activated areas in chemosensory brain regions.

### Materials and methods

### Participants

In this retrospective study, sixty subjects, 27 patients (15 women and 12 men) with long COVID and 33 age-matched healthy controls (20 women and 13 men) were recruited from April 2023 to June 2023. The study was reviewed and approved by the ethics committee “Azienda Ospedaliero Universitaria di Cagliari” (PROT. NP/2023/963) and was performed according to the Declaration of Helsinki. Participants provided their written informed consent to participate in this study. The mean age in the patients’ group was 40.6 ± 13.4 years, with an age range from 22 to 66 years and in healthy controls was 40.5 ± 9.8 years. We enrolled patients with a previous diagnosis of COVID-19 obtained by other specialists or general practitioners suffering from mind fog, fatigue, or persistent chemosensory deficits, also after months from the acute phase of the disease. Inclusion criteria were adult right-handed patients with the continuation of symptoms for at least 3 months after the initial SARS-CoV-2 infection. In the Controls group, we enrolled only subjects never affected by COVID-19.

Exclusion criteria were acute respiratory infections, neurodegenerative diseases, a history of head or neck trauma, chronic rhinitis or rhinosinusitis, asthma, stroke, diabetes, chronic renal disease, and any systemic disease associated with smell disorders. The clinical evaluation of each participant included the following steps: age, sex, weight (kg), height (cm), body mass index (BMI), current medications, smoking history, and employment. In addition, in each subject, olfactory and gustatory function, cognitive abilities, depression level, neurological diseases, and fMRI were evaluated.

### Olfactory and gustatory evaluations

The olfactory function among participants was determined using the Sniffin’ Sticks test (Hummel et al., 1997, 2007). Pens filled with odors were used to deliver the olfactory stimuli. In Sniffin’ Sticks test presentation, the pen’s tip was positioned approximately 2 cm for approximately 3 s in front of both nostrils. Three different olfactory functions were assessed: odor threshold (OT), odor discrimination (OD), and odor identification (OI) (Masala et al., 2018). First, OT was determined for n-butanol with 16 stepwise dilutions. Thresholds were assessed using a single-staircase technique based on a three-alternative forced-choice task (3AFC). Second, OD was assessed over 16 trials. In each discrimination, three pens were presented, two containing the same odor and the third containing the target odorant using the 3AFC task. Third, OI was evaluated using 16 common odors, each presented with four verbal descriptors in a multiple forced-choice format (three distractors and one target). The interval between each odor presentation was 20–30 s. The total score (Threshold + Discrimination + Identification = TDI) was calculated. Scores of ≤ 16, between 16.25 and 30.5, between 30.75 and 41.25, > 41.5 were indicated functional anosmia, hyposmia, normosmia, and supersmellers, respectively (Oleszkiewicz et al., 2019).

The gustatory function was performed by means of the “Taste Strips” test (Burghart Messtechnik, Wedel, Germany). The test consists of filter paper strips impregnated with four concentrations of each basic taste qualities: sweet, bitter, sour, and salty (Landis et al., 2009). Concentrations were: 0.4, 0.2, 0.1, 0.05 g/mL of sucrose for sweet taste; 0.006, 0.0024, 0.0009, 0.0004 g/mL of quinine hydrochloride for bitter taste; 0.3, 0.165, 0.09, 0.05 g/mL of citric acid for sour; 0.25, 0.1, 0.04, 0.016 g/mL of sodium chloride for salty taste (Landis et al., 2009). Drinking water was used as a solvent in each taste modality and to rinse the participant’ mouth before the test. The global gustatory score may range from 0 to 16. A taste score ≥ 9 is considered normogeusia and a score < 9 is classified as hypogeusia (Landis et al., 2009).

### Cognitive ability evaluation

As a cognitive screening test, the Montreal Cognitive Assessment (MoCA) was used, which assesses cognitive impairment in different domains: visual–constructional skills, executive functions, attention and concentration, memory, language, conceptual thinking, calculations, and spatial orientation (Nasreddine et al., 2005; Conti et al., 2015). The total score was 30, and any score ≥ 26 was considered normal.

### Depression level assessment

Depression level was evaluated using the self-reported Beck Depression Inventory (BDI) test (Beck et al., 1961), which includes 21 items with a four-point scale ranging in order of severity from 0 to 3. The depression level was classified as minimal, mild, moderate, and severe, for 0–13,14–19, 20–28, and 29–63, respectively.

### Imaging assessment

The MRI examinations were performed on a 3 Tesla Vantage Titan scanner (Canon Medical Systems, Ōtawara, Japan) with a 32 channels head coil. The MRI protocol included the following sequences for rs-fMRI analysis: (1) 3D-T1-weighted Fast Field Echo (3D-T1 FFE) sequence (echo time = 2.7 ms; repetition time = 5.9 ms, flip angle = 10°; slice thickness = 1 mm; matrix: 256 × 256); (2) T2*-weighted Echo Planar Imaging (T2*-EPI) sequence (echo time = 25 ms; repetition time = 2000 ms; flip angle: 90°; slice thickness: 3.5 mm; matrix: 64 × 88).

The other sequences included in the protocol, i.e. the T2*-weighted Gradient Echoes (GE) (echo time = 9 ms; repetition time = 600 ms; flip angle: 20°; slice thickness: 5 mm; matrix: 320 × 176), the T2-weighted FLuid-Attenuated Inversion Recovery (echo time = 352 ms; repetition time = 7000 ms; flip angle: 90°; slice thickness: 1.5 mm; matrix: 256 × 256), and the 3D-T2-weighted (echo time = 352 ms; repetition time = 6000 ms; flip angle: 90°; slice thickness: 1.5 mm; matrix: 224 × 224). All sequences of the MRI protocol were analyzed by an expert neuroradiologists (M.P., 9 years of radiological experience) to identify intracranial pathological findings such as for example brain tumor and/or congenital anomalies (such as for example partial or complete corpus callosum agenesia). Those subjects with at least one of these conditions were excluded from the final study population.

### Rs-fMRI assessment

The fMRI analysis was made on the Matlab platform vR2020b (Mathworks, Inc., California, USA) with the CONN-fMRI fc toolbox v20b (Whitfield-Gabrieli & Nieto-Castanon, 2012) based on the SPM 12 package (Welcome Department of Imaging Neuroscience, London, UK; http://www.fil.ion.ucl.ac.uk/spm/).

In accordance with previous studies (Porcu et al., 2022a, b), structural 3D-T1 FFE and functional T2* EPI sequences were pre-processed with the CONN’s default pipeline for volume-based analysis. The following steps were implemented: (a) functional realignment and unwarping; (b) slice-timing correction; (c) functional outlier detection with intermediate settings (97^th^ percentile in normative sample in functional outlier detection system: global-signal z-value threshold = 5 standard deviations; subject-motion threshold = 0.9 mm); (d) functional and structural direct segmentation of cerebrospinal fluid (CSF), grey matter, white matter (WM); (e) normalization to Montreal Neurological Institute (MNI) model (Collins et al., 1994) using the default tissue probability maps (structural target resolution = 1 mm; functional target resolution = 2 mm); e) functional smoothing with 8 mm full width half maximum Gaussian kernel filter.

The first ten volumes of T2-weighted EPI sequence were excluded from analysis to limit potential biases derived by the achievement of the steady state magnetization (Porcu et al., 2019, 2021). Subsequently, the following denoising steps were applied to minimize the residual nonneural variability of functional data (Porcu et al., 2021b): (a) linear regression of potential confounding effects, including Blood Oxygen Level Dependent (BOLD) signals recorded in CSF and WM, estimated subject-motion specifications and identified outlier scans for the “scrubbing” procedure; (b) temporal band-pass filtering (0.008 to 0.09 Hz) to decrease noise effects and low frequency drift.

In analogy to previous studies to previous research (Porcu et al., 2021a, 2022a), the fALFF technique was used for the analysis of the processed data by computing for each individual voxel the root mean square of BOLD signal in the low frequency range (0.008 to 0.09 Hz) (Lv et al., 2018; Zou et al., 2008). Brain regions mapping was performed using the CONN’s default atlas: the Harvard-Oxford atlas (Desikan et al., 2006; Tzourio-Mazoyer et al. 2022) for cortical and subcortical regions, and the Automated Anatomical Labelling atlas (TzourioMazoyer et al. 2022) for cerebellar regions (Supplementary material for Table 1).

## Statistical analysis

### Clinical, cognitive, and mood status

Statistical analysis was conducted using Statistical Package for Social Sciences, SPSS 26.0 for Windows (IBM, Armonk, NY, USA).

All data were presented as mean values ± standard deviation (SD). Statistical differences between patients with long COVID symptoms and healthy control groups for all variables were assessed by means of independent sample t test adjusted with Bonferroni correction for multiple comparisons. The values < 0.05 were considered statistically significant.

### Rs-fMRI analyses

Four distinct univariate mass regression fALFF analyses were performed to evaluate odor threshold (Analysis 1), odor discrimination (Analysis 2), odor identification (Analysis 3), and TDI score (Analysis 4) as second-level covariates. Two different statistics (a parametric and a non-parametric one) were applied for every analysis to identify statistically significant results (Nieto-Castanon, 2020):

- Parametric Gaussian Random Field Theory (GRFT) statistic, adopting a cluster level uncorrected p-value (peak p-unc) < 0.001 for height threshold and a size p-value corrected for false discovery rate (size p-FDR) < 0.05 for cluster size threshold (Worsley et al., 1996).
- Non-parametric randomization/permutation statistic, performing 1000 permutation iterations of the original data, adopting a mass p-unc (mass p-unc) < 0.001 for height threshold and size p-FDR < 0.05 as cluster level threshold (Bullmore et al., 1999).

A total of eight sub-analyses were performed: two for Threshold (Sub-analysis 1 A with

GRFT statistics, and Sub-analysis 1B with randomization/permutation statistics), two for Discrimination (Sub-analysis 2 A with GRFT statistics, and Sub-analysis 2B with randomization/permutation statistics), two for Identification (Sub-analysis 3 A with GRFT statistics, and Sub-analysis 3B with randomization/permutation statistics), and two for TDI (Sub-analysis 4 A with GRFT statistics, and Sub-analysis 4B with randomization/permutation statistics). The scheme of the sub-analyses is reported in Table 1.

**Table 1:** Scheme of the fractional amplitude of low frequency fluctuations (fALFF) sub-analyses

Scheme of the fALFF sub-analyses		
fALFF analyses		Statistics
	GRFT	Randomization/permutation
Analysis 1 - Threshold	Sub-analysis 1 A	Sub-analysis 1B
Analysis 2 - Discrimination	Sub-analysis 2 A	Sub-analysis 2B
Analysis 3 - Identification	Sub-analysis 3 A	Sub-analysis 3B
Analysis 4 - TDI score	Sub-analysis 4 A	Sub-analysis 4B

## Results

### Study population

The Table 2 indicated mean values ± standard deviation of olfactory perception (threshold, discrimination, identification, and their sum TDI score), gustatory function (sweet, salty, sour, bitter, and their sum), cognitive abilities (MoCA), and depression scale (BDI-II). In our study no significant differences were observed for age, height, weight, and BMI (Table 2).

**Table 2:** Demographic and clinical information of all participants. Data are expressed as mean ± SD

	Patients			Control		*p* Value
Demographics	*N* = 27			*N* = 33		
Sex N (% female)		15 (55.6%)		20 (58.8%)		0.802
Age	40.6 ± 13.4			40.5 ± 9.8		0.993
Weight (Kg)	71.0 ± 21.1			64.4 ± 12.8		0.138
Height (cm)	166.8 ± 10.7			165.8 ± 8.1		0.673
BMI	25.3 ± 6.5			23.3 ± 4.0		0.152
Sweet	3.4 ± 1.1			3.4 ± 0.9		0.791
Salty	3.4 ± 0.9			3.2 ± 1.1		0.519
Sour	2.6 ± 1.1			2.7 ± 0.9		0.551
Bitter	2.4 ± 1.5			3.1 ± 1.2		**< 0.05**
Total taste	11.7 ± 3.8			12.5 ± 2.2		0.327
MoCA	27.6 ± 2.6			27.9 ± 1.8		0,631
BDI-II	11.4 ± 9.7			6.6 ± 5.5		**< 0.02**

Legend: BDI-II = Beck Depression Inventory; BMI = body mass index; SD = standard deviation; OT = odor threshold; OD = odor discrimination; OI = odor identification; TDI score = threshold, discrimination, and identification score; MoCA = Montreal Cognitive Assessment. Significant p values are highlighted in bold

**Fig. 1:**
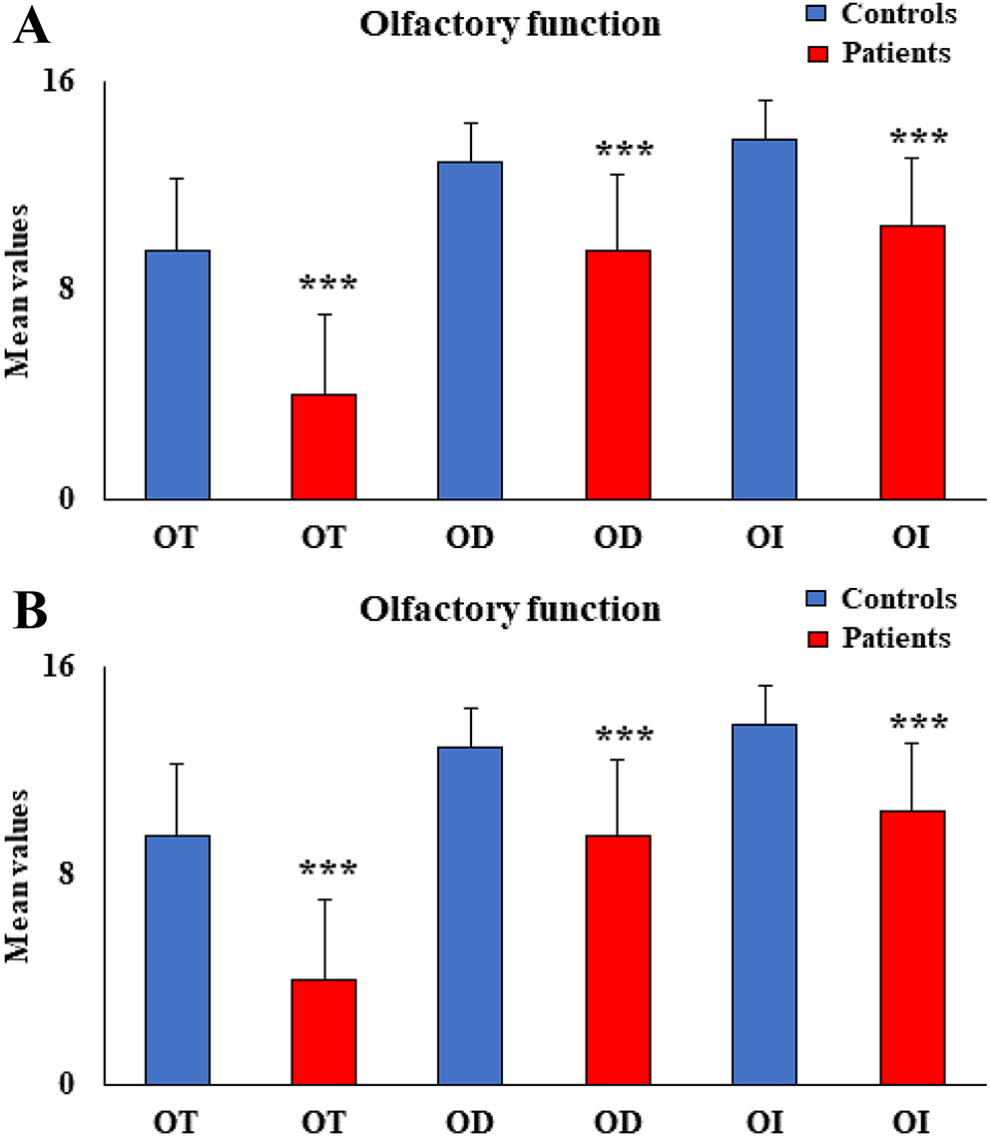
(**A**) Mean values ± standard deviation (SD, vertical bars) of odor threshold (OT), odor discrimination (OD), odor identification (OI) for patients with long COVID symptoms (*n* = 27) compared to healthy controls (*n* = 276 33). ***p ≤ 0.001. (**B**) Mean values ± standard deviation (SD, vertical bars) of the TDI score (threshold, discrimination, and identification sum) for patients with long COVID symptoms (*n* = 27) compared to healthy controls (*n* = 33). ***p ≤ 0.001

Instead, patients with long COVID symptoms showed a significant decrease compared to healthy controls in all olfactory function parameters such as OT [F_(1,60)_ = 52.683, *p* < 0.001, partial η^2^ = 0.472], OD [F_(1,60)_ = 34.600, *p* < 0.001, partial η^2^ = 0.370], OI [F_(1,60)_ = 38.062, *p* < 0.001, partial η^2^ = 0.392](Fig. 1A), and TDI score [F_(1,60)_ = 98.117, *p* < 0.001, partial η^2^ = 0.624] (Fig. 1B). In patients with long COVID, mean values ± standard deviation were 4.0 ± 3.1, 9.5 ± 2.9, 10.5 ± 2.6, and 24.1 ± 7.1 for OT, OD, OI, and TDI score, respectively. In healthy controls, mean values ± standard deviation were 9.5 ± 2.8, 12.9 ± 1.5, 13.8 ± 1.5, 36.2 ± 1.4 for OT, OD, 270 OI, and TDI score, respectively.

In the group of patients with long COVID symptoms as regards olfactory function were found the following percentages for anosmia, hyposmia, and normosmia, 7% (*n* = 2), 74% (*n* = 20), and 19% (*n* = 5), respectively, while in the age-matched healthy controls group all subjects showed normosmia.

As regards gustatory function no significant differences (*p* > 0.05) were observed between patients with long COVID and healthy controls for sweet, salty, sour, and global taste perception (Table 2). Instead, a significant difference (*p* < 0.05) between patients with long COVID and healthy controls was found only for bitter taste perception, mean value ± standard deviation was 2.4 ± 1.5 and 3.1 ± 1.2 in patients with long COVID and healthy controls, respectively. Among patients with long COVID only 30% (*n* = 8) of the subjects exhibited hypogeusia and 70% (*n* = 19) showed normogeusia, while in controls group only 3% (*n* = 1) patients showed hypogeusia and 97% (*n* = 33) of subjects had normosmia.

In the cognitive abilities (MoCA) no significant differences (*p* > 0.05) were observed between patients with long COVID and healthy controls. However, in the cognitive evaluations we observed that 22% (*n* = 6) showed a mild cognitive impairment with a global score around 25. As regards depression level, patients with long COVID symptoms showed a significant increase in depression scores compared to healthy controls [F_(1,59)_ = 5.762, *p* < 0.05, partial η^2^ = 0.089]. In patients with long COVID the percentages for minimal, mild, moderate, and severe depression level, were 78% (*n* = 21), 5% (*n* = 1), 11% (*n* = 3), and 7% (*n* = 2), respectively.

Finally, we focused our attention to analyze in patients with long COVID symptoms the correlation between the olfactory test perception and the volume of activated areas in chemosensory brain regions. The check of the MRI images by the expert neuroradiologist evidence no pathological findings for all the subjects of the study population, and for this reason none of them was excluded from the study. In patients with long COVID symptoms the most prevalent symptoms in COVID-19 acute phase were fever (74%), dry cough or cough with mucus (56%), and muscle or joint pain (33%).

### Resting-state functional MRI

The quality control data of the study population following the pre-processing step are reported in Supplementary material for Table 3. Of the eight sub-analyses, sub-analysis 2 A (second-level covariate: odor discrimination; statistic: GRFT), 4 A (second-level covariate: TDI score; statistics: GRFT) and 4B (second-level covariate: TDI score; statistics: permutation/randomization) showed statistically significant results. No statistically significant findings were found in the other sub-analyses. Sub-analysis 2 A revealed a single cluster of 341 voxels of increased fALFF (peak p-unc < 0.001; size p-FDR < 0.001), located in the right hemisphere in the frontal pole and in the superior frontal gyrus for the odor discrimination (Table 3).

**Table 3:** Results of sub-analysis 2 A

Results of sub-analysis 2 A (second level covariate: discrimination; statistics: GRFT)		
Cluster coordinates (x, y, z) according to the MNI space (mm)	Size size p-FWE size p-FDR size p-unc peak p-FWE	peeak p-unc
18 + 52 + 32	341 < 0.001 < 0.001 < 0.001 0.654	< 0.001
	Voxels that showed statistically significant increased fALFF	
219 voxels (64%) covering 3% of atlas. FP r (Frontal Pole Right)		
94 voxels (28%) covering 4% of atlas. SFG r (Superior Frontal Gyrus Right)		
28 voxels (8%) covering 0% of atlas. not-labeled		

Legend: GRFT = Gaussian Random Field Theory; MNI = Montreal Neurological Institute; p-FWE = p-value

corrected for Family Wise Error; p-FDR = p-value corrected for False discovery rate; p-unc = uncorrected p-value; 321 fALFF = fractional Amplitude of Low Frequency Fluctuations

Sub-analysis 4 A revealed a single cluster of 192 voxels of increased fALFF (peak p-unc < 324 0.001; size p-FDR = 0.006), located, also in this case, in the right frontal pole and in the right superior frontal gyrus for the TDI score (Table 4).

**Table 4:** Results of sub-analysis 4 A

Results of sub-analysis 4 A (second level covariate: TDI score; statistics: GRFT)	
Cluster coordinates (x, y, z) according to Size size p-FWE size p-FDR size p-unc peak p-FWE the MNI space (mm)	peeak p-unc
18 + 52 + 32 192 0.005 0.006 < 0.001 0.816	< 0.001
Voxels that showed statistically significant increased fALFF	
180 voxels (94%) covering 2% of atlas. FP r (Frontal Pole Right)	
2 voxels (1%) covering 0% of atlas. SFG r (Superior Frontal Gyrus Right)	
10 voxels (5%) covering 0% of atlas.not-labeled	

Legend: TDI score = threshold, discrimination, and identification score; GRFT = Gaussian Random Field Theory; MNI = Montreal Neurological Institute; p-FWE = p-value corrected for Family Wise Error; p-FDR = p-value corrected for False discovery rate; p-unc = uncorrected p-value; fALFF = fractional Amplitude of Low Frequency Fluctuations. Atlas – ROIs legends (Desikan et al., 2006; Tzourio-Mazoyer et al., 2002)

Sub-analysis 4B revealed a single cluster of 1938 voxels of increased fALFF (mass p-unc < 0.001; size p-FDR = 0.022), located in the frontal pole and superior frontal gyrus of both hemispheres, and in the right middle frontal gyrus for the TDI score (Table 5).

**Table 5:** Results of sub-analysis 4B

Results of sub-analysis 4B (second level covariates: TDI; statistics: permutation / randomization)			
Cluster coordinates (x, y, z) Size size p-FWE size p-FDR size p-unc mass mass p-FWE mass p-FDR according to the MNI space (mm)			mass punc
18 + 52 + 32 1938 0.021 0.022 < 0.001 21999.05 0.023	0.024	< 0.001	
Voxels that showed statistically significant increased fALFF			
940 voxels (49%) covering 12% of atlas. FP r (Frontal Pole Right)			
498 voxels (26%) covering 19% of atlas. SFG r (Superior Frontal Gyrus Right)			
180 voxels (9%) covering 6% of atlas. SFG l (Superior Frontal Gyrus Left)			
81 voxels (4%) covering 1% of atlas. FP l (Frontal Pole Left)			
32 voxels (2%) covering 1% of atlas. MidFG r (Middle Frontal Gyrus Right)			
207 voxels (11%) covering 0% of atlas.not-labeled			

Legend: TDI score = threshold, discrimination, and identification score; MNI = Montreal Neurological Institute; p-FWE = p-value corrected for Family Wise Error; p-FDR = p-value corrected for False discovery rate; p-unc = uncorrected p-value; fALFF = fractional Amplitude of Low Frequency Fluctuations. Atlas – ROIs legends (Desikan et al., 2006; Tzourio-Mazoyer et al., 2002)

**Fig. 2:**
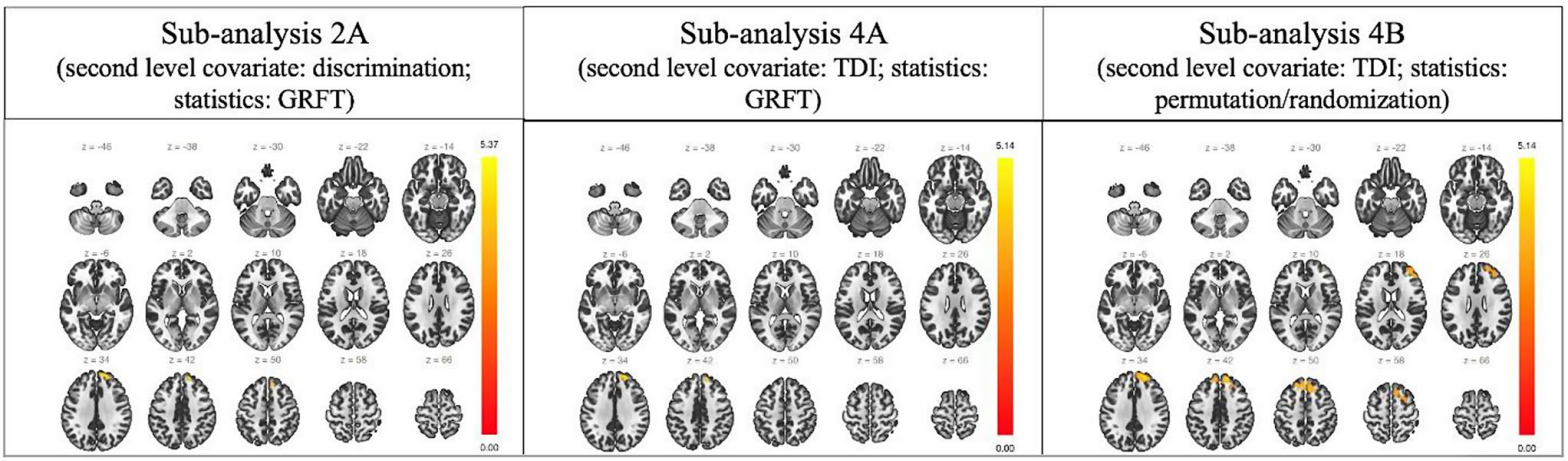
Graphic representation of the results of sub-analyses **2 A**, **4 A**, and **4B** according to a slices scheme on the axial (z) plane and the coordinates on the MNI scheme. Only clusters of increased fALFF were identified (yellowish/reddish)

**Fig. 3:**
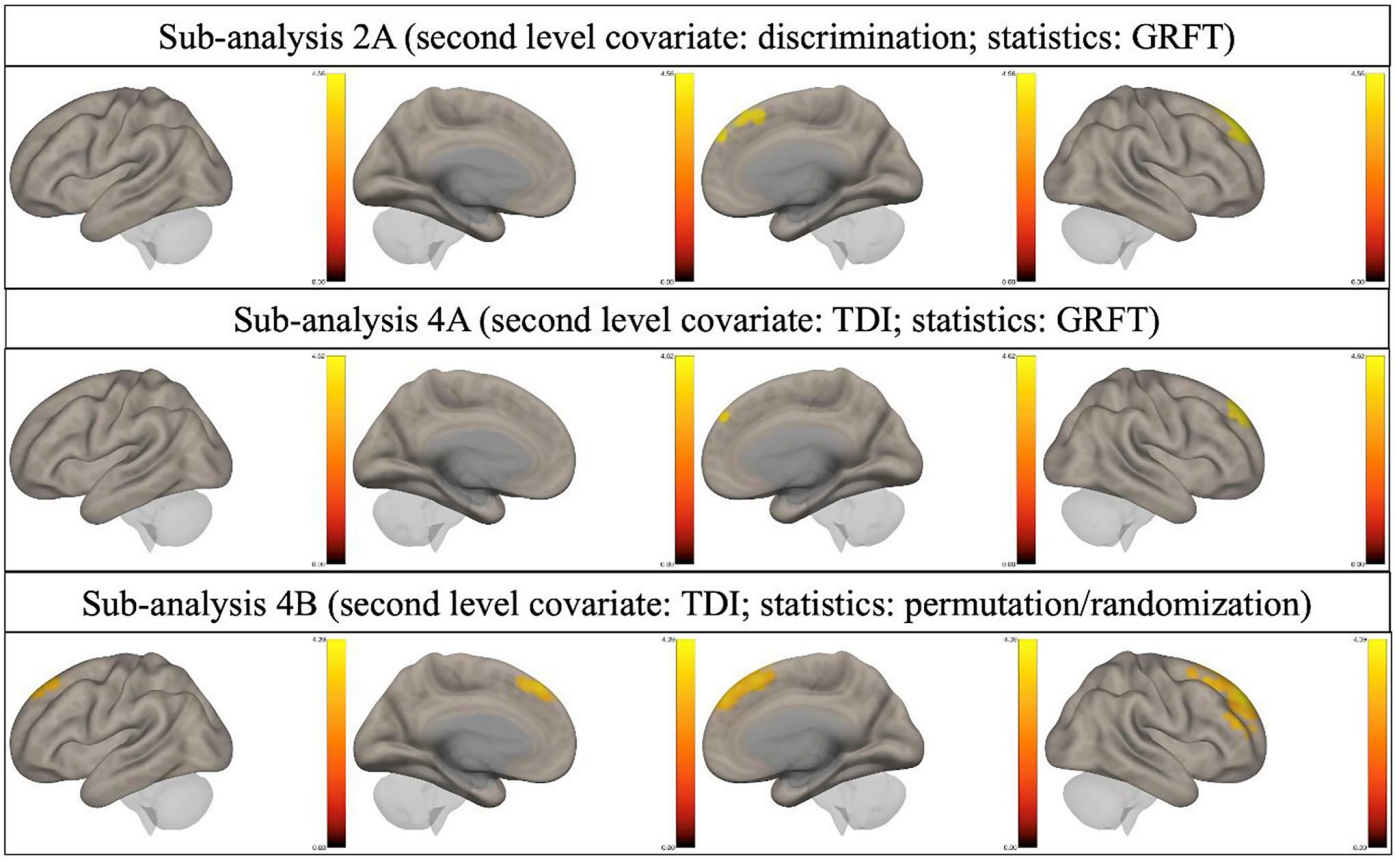
Three-dimensional graphic representation of the results of sub-analyses **2 A**, **4 A**, and **4B**. Only clusters of increased fALFF were identified (yellowish/reddish)

No clusters of reduced fALFF were found in all the above-mentioned sub-analyses for odor 343 threshold, odor identification, cognitive abilities, and depression level. A graphical representation of results is reported in Figs. 2 and 3.

## Discussion

Our data showed that patients with long COVID symptoms exhibited a significant decrease in odor threshold, odor discrimination, odor identification, and in their sum TDI score compared to age-matched healthy controls. Among patients with long sequelae of COVID-19, the following percentages were observed: 7% and 74% for the anosmia and hyposmia respectively, obtained using psychophysical olfactory tests. The presence of these olfactory deficits in patients with long COVID supports the idea of neuronal damage in brain pathways. In addition, among patients with long COVID, only 30% (*n* = 8) of subjects exhibited hypogeusia as indicated in a previous study (Hintschich et al., 2022).

In this study, we adopted the fALFF technique as a functional segregation method for the analysis of rs-fMRI data instead of other functional integration approaches, such as seed-based connectivity. Our aim was to assess differences in regional neural activity related to olfactory test performance rather than differences in functional connectivity, as was done, for example, by Wingrove et al. (Wingrove et al., 2023). Additionally, we chose the fALFF technique because it has been reported in the literature that this method is more specific to grey matter compared to other techniques, such as ALFF (Lv et al., 2018; Zou et al., 2008).

Interestingly, our data using the rs-fMRI, which is an innovative and alternative technique to study the functional connectivity (FC), showed that in patients with long sequelae of COVID-19, the odor discrimination activated the right hemisphere in the frontal pole and in the superior frontal gyrus. Our study is focused on patients with long sequelae of COVID-19, since a previous study (Muccioli et al., 2023), evaluated the neuropsychological profile and the integrity of the olfactory network in patients with long sequelae of COVID-19 compared to healthy controls. Our data, in line with this previous study (Muccioli et al., 2023) did not observe morphological alterations in patients with long sequelae of COVID-19. In addition, a previous study in healthy controls suggested that individual differences in olfactory behavior are encoded by diverse structural network fingerprints in the olfactory cortex (Fjaeldstad et al., 2021). Our data showed that a better score in olfactory function may be correlated to an increased number of voxels activated in the fMRI study. This finding suggests a direct correlation between olfactory ability, the number of fibers, and the volumetric measures of the olfactory sulcus, olfactory bulb, and the structure of the orbitofrontal cortex (Buschhuter et al. 2008; Frosolini et al., 2022; Hummel et al., 2003).

The pathophysiology underlying long COVID clinical manifestations is still debated. Our results suggested the existence of both central and peripheral impairments in the nervous system during long COVID clinical manifestations, as indicated in previous studies (Carfi et al. 2020; Huang et al., 2021; Pilotto et al., 2021; Xu et al., 2022; Thomasson et al., 2023; Wingrove et al., 2023), since the odor threshold is more dependent on the nasal anatomy and the number of receptors expressed in the olfactory epithelium (Hummel et al., 2017; Masala et al., 2019). Instead, odor discrimination and identification are more related to central processes such as the anterior olfactory nucleus, olfactory tubercle, piriform cortex, amygdala, and orbitofrontal cortex (Gottfried, 2010).

Our results indicated significant correlations between odor discrimination and increased activation in the right hemisphere, in the frontal pole, and in the superior frontal gyrus. However, the brain activation in fMRI studies may be related to the type of odor perceived and to the pleasantness, since a previous study suggested increased activation in the right angular gyrus in response to well-being odors such as “musk flower” and “orange”, and increased activity in the left angular gyrus in response to neutral stimuli (Joshi et al., 2023). Instead, a previous study suggested activation of the left anterior insula, frontopolar and middle frontal gyrus during the odor discrimination (Plailly et al., 2007). The left anterior insula is usually involved in odor property evaluation, while the activation of the frontopolar and middle frontal gyrus is more related to working memory during the odor discrimination task (Plailly et al., 2007).

Moreover, we also observed significant correlations in both analyses (GRFT and permutation/randomization) between global olfactory function (TDI score) and the activity of the frontal pole and superior frontal gyrus in both hemispheres and in the right middle frontal gyrus. The absence of a statistical correlation between the odor threshold and brain area activation was predictable because it is well known that the odor threshold is more related to a peripheral function of the olfactory system and the number of receptors expressed in the nasal epithelium. This finding suggests different levels of hierarchically organized networks for odor discrimination and odor identification, as previously reported (Hedner et al., 2010; Savic et al., 2000). Interestingly, the significant correlations in odor discrimination but no in odor identification suggested that the odor identification is probably related to a specific network between sensory and cognitive/memory areas of the brain, such as the cingulate cortex, insula, frontal pole, and hippocampus. Instead, the odor identification may activate other brain areas, such as the entorhinal cortex, which plays a connection between the hippocampus and temporal cortex with a key role in memory retrieval. Our results suggest that odor discrimination and odor identification could indicate distinct representations of the odor processing hierarchy, since the odor discrimination may be an early stage of the odor processing, while odor identification is considered a later stage, as indicated by Cormiea and Fischer (2023). Based on this finding, it is likely that in humans, the odor discrimination function is always activated, explaining the results obtained in rs-fMRI with the activation of the right frontal area also in resting state. Instead, odor identification constitutes a second stage in the odor processing hierarchy, requiring the activation of the knowledge and memory systems.

The connection between olfaction and emotions originates from our complex neuroanatomical structure. It is hypothesized that certain odor classes, closely linked to specific emotional responses, significantly impact distinct cerebral networks. This correlation, rooted in our neurobiological framework, explains the varying resilience of these networks in different circumstances. In fact, the rs-fMRI is a condition of vigilance and lucidity in the subject that does not require concentration, but is immersed in a quantity of environmental stimuli, including the olfactory ones constantly present in the environment. The finding of activation in the right hemisphere in the frontal pole and in the superior frontal gyrus may be interpreted as involvement of the dorsal attention network for the external stimuli present in the environment due to the constant attention of the subject awake.

Limitations of this study may include the absence of severity stratification in patients with long COVID, the low number of patients recruited, and the absence of specific SARS-CoV-2 strain identification for each patient.

## Conclusions

This study indicated that olfactory dysfunction is an important chronic symptom in patients with long COVID-19 associated with central nervous system impairment. Our data indicated that the rs-fMRI in combination with the objective evaluation of olfactory and gustatory function may be useful for the evaluation of patients with long COVID associated to anosmia and hyposmia. Our results showed significant correlations between odor discrimination and the increased activation in the right hemisphere, in the frontal pole, and in the superior frontal gyrus. However, to reach a therapeutic goal in patients with long COVID it is necessary to use a multidisciplinary approach that may provide a neurological, physiological, and imaging assessment of patients.

## Electronic supplementary material

Below is the link to the electronic supplementary material.

Supplementary Material 1

Supplementary Material 2

## Data Availability

The datasets generated and analyzed during the current study are available from the corresponding author on reasonable request.

## Abbreviations

3D-T1 FFE: 3D-T1-weighted Fast Field Echo
BDI-II: Beck Depression Inventory
BOLD: Blood oxygen level dependent
COVID-19: coronavirus disease 2019
CSF: Cerebrospinal fluid
fALFF: Amplitude of Low Frequency Fluctuations
FC: functional connectivity
GRFT: Gaussian Random Field Theory
OT: Odor threshold
OD: Odor discrimination
OI: Odor identification
MoCA: Montreal Cognitive Assessment
SD: Standard deviations
T2*-EPI: T2*-weighted Echo Planar Imaging
TDI score: Threshold + Discrimination + Identification
WM: White matter
