# Supplementary material for: Neuroimaging evaluations of olfactory, gustatory, and neurological deficits in patients with long-term sequelae of COVID-19
Source: Brain Imaging Behav. 2024 Sep 28;18(6):1480–90. doi: 10.1007/s11682-024-00936-0 (PMC11680602; doi:10.1007/s11682-024-00936-0)
Supplement: Supplementary file 1 — Supplementary Material 1 [file 11682_2024_936_MOESM1_ESM.docx]

**Supplementary Table 1**

Atlas – ROIs legends (Desikan et al., 2006; Tzourio-Mazoyer et al., 2002)

- Atlas: FP r = Frontal Pole Right
- FP l = Frontal Pole Left
- IC r = Insular Cortex Right
- IC l = Insular Cortex Left
- SFG r = Superior Frontal Gyrus Right
- SFG l = Superior Frontal Gyrus Left
- MidFG r = Middle Frontal Gyrus Right
- MidFG l = Middle Frontal Gyrus Left
- IFG tri r = Inferior Frontal Gyrus, pars triangularis Right
- IFG tri l = Inferior Frontal Gyrus, pars triangularis Left
- IFG oper r = Inferior Frontal Gyrus, pars opercularis Right
- IFG oper l = Inferior Frontal Gyrus, pars opercularis Left
- PreCG r = Precentral Gyrus Right
- PreCG l = Precentral Gyrus Left
- TP r = Temporal Pole Right
- TP l = Temporal Pole Left
- aSTG r = Superior Temporal Gyrus, anterior division Right
- aSTG l = Superior Temporal Gyrus, anterior division Left
- pSTG r = Superior Temporal Gyrus, posterior division Right
- pSTG l = Superior Temporal Gyrus, posterior division Left
- aMTG r = Middle Temporal Gyrus, anterior division Right
- aMTG l = Middle Temporal Gyrus, anterior division Left
- pMTG r = Middle Temporal Gyrus, posterior division Right
- pMTG l = Middle Temporal Gyrus, posterior division Left
- toMTG r = Middle Temporal Gyrus, temporooccipital part Right
- toMTG l = Middle Temporal Gyrus, temporooccipital part Left
- aITG r = Inferior Temporal Gyrus, anterior division Right
- aITG l = Inferior Temporal Gyrus, anterior division Left
- pITG r = Inferior Temporal Gyrus, posterior division Right
- pITG l = Inferior Temporal Gyrus, posterior division Left
- toITG r = Inferior Temporal Gyrus, temporooccipital part Right
- toITG l = Inferior Temporal Gyrus, temporooccipital part Left
- PostCG r = Postcentral Gyrus Right
- PostCG l = Postcentral Gyrus Left
- SPL r = Superior Parietal Lobule Right
- SPL l = Superior Parietal Lobule Left
- aSMG r = Supramarginal Gyrus, anterior division Right
- aSMG l = Supramarginal Gyrus, anterior division Left
- pSMG r = Supramarginal Gyrus, posterior division Right
- pSMG l = Supramarginal Gyrus, posterior division Left
- AG r = Angular Gyrus Right
- AG l = Angular Gyrus Left
- sLOC r = Lateral Occipital Cortex, superior division Right
- sLOC l = Lateral Occipital Cortex, superior division Left
- iLOC r = Lateral Occipital Cortex, inferior division Right
- iLOC l = Lateral Occipital Cortex, inferior division Left
- ICC r = Intracalcarine Cortex Right
- ICC l = Intracalcarine Cortex Left
- MedFC = Frontal Medial Cortex
- SMA r = Juxtapositional Lobule Cortex -formerly Supplementary Motor Cortex- Right
- SMA L= Juxtapositional Lobule Cortex -formerly Supplementary Motor Cortex- Left
- SubCalC = Subcallosal Cortex
- PaCiG r = Paracingulate Gyrus Right
- PaCiG l = Paracingulate Gyrus Left
- AC = Cingulate Gyrus, anterior division
- PC = Cingulate Gyrus, posterior division
- Precuneus = Precuneous Cortex
- Cuneal r = Cuneal Cortex Right
- Cuneal l = Cuneal Cortex Left
- FOrb r = Frontal Orbital Cortex Right
- FOrb l = Frontal Orbital Cortex Left
- aPaHC r = Parahippocampal Gyrus, anterior division Right
- aPaHC l = Parahippocampal Gyrus, anterior division Left
- pPaHC r = Parahippocampal Gyrus, posterior division Right
- pPaHC l = Parahippocampal Gyrus, posterior division Left
- LG r = Lingual Gyrus Right
- LG l = Lingual Gyrus Left
- aTFusC r = Temporal Fusiform Cortex, anterior division Right
- aTFusC l = Temporal Fusiform Cortex, anterior division Left
- pTFusC r = Temporal Fusiform Cortex, posterior division Right
- pTFusC l = Temporal Fusiform Cortex, posterior division Left
- TOFusC r = Temporal Occipital Fusiform Cortex Right
- TOFusC l = Temporal Occipital Fusiform Cortex Left
- OFusG r = Occipital Fusiform Gyrus Right
- OFusG l = Occipital Fusiform Gyrus Left
- FO r = Frontal Operculum Cortex Right
- FO l = Frontal Operculum Cortex Left
- CO r = Central Opercular Cortex Right
- CO l = Central Opercular Cortex Left
- PO r = Parietal Operculum Cortex Right
- PO l = Parietal Operculum Cortex Left
- PP r = Planum Polare Right
- PP l = Planum Polare Left
- HG r = Heschl's Gyrus Right
- HG l = Heschl's Gyrus Left
- PT r = Planum Temporale Right
- PT l = Planum Temporale Left
- SCC r = Supracalcarine Cortex Right
- SCC l = Supracalcarine Cortex Left
- OP r = Occipital Pole Right
- OP l = Occipital Pole Left
- Thalamus r = Thalamus Right
- Thalamus l = Thalamus Left
- Caudate r = Caudate Right
- Caudate l = Caudate Left
- Putamen r = Putamen Right
- Putamen l = Putamen Left
- Pallidum r = Pallidum Right
- Pallidum l = Pallidum Left
- Hippocampus r = Hippocampus Right
- Hippocampus l = Hippocampus Left
- Amygdala r = Amygdala Right
- Amygdala l = Amygdala Left
- Accumbens r = Accumbens Right
- Accumbens l = Accumbens Left
- Brain-Stem = Brain Stem
- Cereb1 l = Cerebelum Crus1 Left
- Cereb1 r = Cerebelum Crus1 Right
- Cereb2 l = Cerebelum Crus2 Left
- Cereb2 r = Cerebelum Crus2 Right
- Cereb3 l = Cerebelum 3 Left
- Cereb3 r = Cerebelum 3 Right
- Cereb45 l = Cerebelum 4 5 Left
- Cereb45 r = Cerebelum 4 5 Right
- Cereb6 l = Cerebelum 6 Left
- Cereb6 r = Cerebelum 6 Right
- Cereb7 l = Cerebelum 7b Left
- Cereb7 r = Cerebelum 7b Right
- Cereb8 l = Cerebelum 8 Left
- Cereb8 r = Cerebelum 8 Right
- Cereb9 l = Cerebelum 9 Left
- Cereb9 r = Cerebelum 9 Right
- Cereb10 l = Cerebelum 10 Left
- Cereb10 r = Cerebelum 10 Right
- Ver12 = Vermis 1 2
- Ver3 = Vermis 3
- Ver45 = Vermis 4 5
- Ver6 = Vermis 6
- Ver7 = Vermis 7
- Ver8 = Vermis 8
- Ver9 = Vermis 9
- Ver10 = Vermis 10
