# Supplementary material for: Neuroimaging evaluations of olfactory, gustatory, and neurological deficits in patients with long-term sequelae of COVID-19
Source: Brain Imaging Behav. 2024 Sep 28;18(6):1480–90. doi: 10.1007/s11682-024-00936-0 (PMC11680602; doi:10.1007/s11682-024-00936-0)
Supplement: Supplementary file 2 — Supplementary Material 2 [file 11682_2024_936_MOESM2_ESM.docx]

| **Subjects** | **Age** | **Sex (Male = 0; Female = 1)** | **Valid scans** | **Invalid scans** | **Max motion (mm)** | **Mean motion (mm)** | **Max global signal change (std)** | **Mean global signal change (std)** | **BOLD signal after denoising (std)** | **Global correlation at rest** |
| --- | --- | --- | --- | --- | --- | --- | --- | --- | --- | --- |
| **1** | 24 | 0 | 236 | 4 | 0.531 | 0.121 | 241.727 | 0.860 | 0.447 | 0.007 |
| **2** | 49 | 1 | 233 | 7 | 1.229 | 0.097 | 145.034 | 0.705 | 0.460 | 0.007 |
| **3** | 56 | 0 | 183 | 57 | 7.598 | 0.470 | 80.375 | 0.780 | 0.738 | 0.009 |
| **4** | 22 | 0 | 230 | 10 | 1.598 | 0.181 | 42.478 | 0.794 | 1.103 | 0.011 |
| **5** | 66 | 1 | 231 | 9 | 0.812 | 0.200 | 591.360 | 0.756 | 0.551 | 0.012 |
| **6** | 22 | 1 | 229 | 11 | 1.199 | 0.131 | 163.128 | 0.807 | 0.525 | 0.006 |
| **7** | 35 | 1 | 237 | 3 | 0.983 | 0.143 | 160.550 | 0.778 | 0.547 | 0.007 |
| **8** | 44 | 1 | 237 | 3 | 0.788 | 0.151 | 115.946 | 0.763 | 0.644 | 0.020 |
| **9** | 33 | 0 | 238 | 2 | 0.863 | 0.169 | 107.301 | 0.823 | 0.778 | 0.018 |
| **10** | 38 | 1 | 234 | 6 | 1.514 | 0.151 | 98.831 | 0.812 | 0.602 | 0.017 |
| **11** | 44 | 1 | 238 | 2 | 1.292 | 0.197 | 50.825 | 0.779 | 0.758 | 0.011 |
| **12** | 40 | 0 | 231 | 9 | 2.461 | 0.223 | 88.472 | 0.766 | 0.681 | 0.009 |
| **13** | 47 | 0 | 234 | 6 | 1.155 | 0.176 | 55.388 | 0.767 | 0.800 | 0.011 |
| **14** | 42 | 0 | 237 | 3 | 0.823 | 0.147 | 81.893 | 0.709 | 0.500 | 0.006 |
| **15** | 35 | 1 | 232 | 8 | 1.124 | 0.147 | 206.549 | 0.811 | 0.432 | 0.009 |
| **16** | 43 | 1 | 234 | 6 | 1.205 | 0.177 | 182.796 | 0.789 | 0.479 | 0.007 |
| **17** | 23 | 1 | 232 | 8 | 1.392 | 0.157 | 127.785 | 0.765 | 0.560 | 0.008 |
| **18** | 54 | 0 | 225 | 15 | 3.967 | 0.268 | 64.044 | 0.731 | 0.864 | 0.009 |
| **19** | 66 | 0 | 235 | 5 | 1.008 | 0.395 | 114.181 | 0.866 | 0.688 | 0.007 |
| **20** | 28 | 1 | 233 | 7 | 1.211 | 0.321 | 161.711 | 0.979 | 0.742 | 0.011 |
| **21** | 23 | 1 | 238 | 2 | 0.553 | 0.096 | 132.614 | 0.763 | 0.448 | 0.009 |
| **22** | 65 | 1 | 237 | 3 | 0.677 | 0.357 | 243.073 | 0.781 | 0.558 | 0.010 |
| **23** | 32 | 1 | 237 | 3 | 1.067 | 0.109 | 86.135 | 0.802 | 0.626 | 0.007 |
| **24** | 46 | 1 | 236 | 4 | 1.340 | 0.117 | 218.350 | 0.790 | 0.407 | 0.006 |
| **25** | 45 | 0 | 224 | 16 | 1.822 | 0.162 | 145.305 | 0.903 | 0.684 | 0.011 |
| **26** | 47 | 0 | 237 | 3 | 0.813 | 0.257 | 115.141 | 0.777 | 0.573 | 0.013 |
| **27** | 26 | 0 | 235 | 5 | 2.162 | 0.158 | 46.622 | 0.738 | 0.446 | 0.012 |

**Supplementary table 2: Quality control data of rs-fMRI data following preprocessing.** BOLD = Blood oxygen level dependent; std = standard deviation.
